# Design and Development of an E-Textile Mat for Assuring the Comfort of Bedridden Persons

**DOI:** 10.3390/ma14185437

**Published:** 2021-09-20

**Authors:** Daniela Sofronova, Radostina A. Angelova, Yavor Sofronov

**Affiliations:** 1 Centre of Competence MIRACle—Mechatronics, Innovation, Robotics, Automation, Clean Technologies, Laboratory “Intelligent Mechatronic Solutions in the Field of Textiles and Clothing”, Technical University of Sofia, 1000 Sofia, Bulgaria; dcholeva@tu-sofia.bg; 2 Laboratory “CAD/CAM/CAE in Industry”, Technical University of Sofia, 1000 Sofia, Bulgaria; ysofronov@tu-sofia.bg

**Keywords:** e-textiles, health care, multifunctional textiles, bedridden persons, decubitus ulcers, human comfort, body position, textile sensors, conductive threads, monitoring system

## Abstract

An e-textile mat with capacitive textile sensors was designed and manufactured to monitor body position and prevent decubitus ulcers in the case of bedridden people. The sensors were incorporated through a process of machine embroidery with electrically conductive threads. A new production method for the conductive threads is still expected to be developed, resulting in good conductive properties, high wear resistance and durability. Samples of five variants of motifs without cross-stitching were studied, and the capacity and electrical resistance were determined experimentally. A prototype of the e-textile mat was made with a motif showing the best ratio between the inserted thread and the measured capacity. A hardware solution and a software application for collecting, processing and visualising the received information were developed. Tests were performed in real conditions, which clearly showed that the designed e-textile mat could be successfully applied for non-invasive and continuous control of the position of the human body in a supine position to prevent decubitus ulcers.

## 1. Introduction

Decubitus ulcers are a severe medical problem that is difficult to treat. They are usually the result of some tissue nutrition disorder; the most common cause is lying sick for a long time. In places of contact of the body with a hard pad (mattress), the perfusion with the tissue’s blood worsens. As a result, its nutrition hampers, leading to the appearance of necrosis and concomitant wound [1]. The main principle for avoiding decubitus ulcers is prevention, which is expressed in frequent changes in the body position of a bedridden patient or the use of an anti-decubitus mattress.

The firmness of the mattress, temperature and humidity are the main physico-mechanical and physiological factors that affect the quality of sleep and the comfort of the body lying in bed [2]. The use of e-textiles with sensors to monitor body pressure on the mattress has been investigated in [3,4]. However, the pressure sensors were used to provide information on the areas with the most active wear for each person, depending on the weight, gender and sleeping position as well as for the improvement of the mattress’ ergonomics and sleeping quality.

At present, there are many examples of developed textile sensors [5,6,7,8,9,10,11,12,13], but few are used to monitor the distribution of pressure and, respectively, hardness, in the process of operation. In [5], some commercially available systems and devices for beds and cushions were discussed. It should be mentioned, however, that the price impedes the large-scale application of these products. The authors of [10] presented technology for smart jacket production. In [12], the possibility of measuring the sitting posture via textile pressure sensors was investigated. A new resistive pressure sensing principle was applied in [6]. The authors used conductive yarns and different technologies, such as embroidery, hand sewing, and weaving, for textile sensor production. Textile capacitive pressure sensors were developed in [7,8]. In [7], foam was used as a layer between the electrodes. In [8], the 3D compressible spacer fabric of Mueller Textil, Germany, was applied, the compressibility of which affected the sensor’s performance. Some examples for smart textiles production using the embroidery technique are described in [9]. Detailed analyses of the methods for obtaining textile pressure control sensors are presented in [14,15].

Various technologies are used to produce e-textiles with sensors, one of which is machine embroidery of electrically conductive threads [16]. The main advantage of this technology is that it can change the functionality of a finished traditional textile product in a less expensive, labour-consuming and time-consuming process.

Our study was dedicated to designing and developing an e-textile mat with textile sensors, incorporated by embroidery, applied for the movement monitoring of bedridden people. The continuous data from sensors would give information about the period of immobility of the individual parts of the body. As a result, the position of a bedridden person could be regularly changed in situations where a caregiver is not necessarily present at the bedside or when the medical staff shifts. Communications and programs are also built to collect and process the data from the sensors and provoke reactions. The designed e-textile mat could have a great social impact.

## 2. Materials and Methods

### 2.1. The Design Idea

The design idea was to incorporate textile capacitive sensors in a thin mat, which ought to be set under the body of the bedridden person. The sensors should have the ability to register the movement of the body. The designed mat does not involve the zone of the head, as, usually, there is a pillow under the head.

The selection of the structure of the e-textile mat was carried out in three main directions:

- Selection of the sensors and measuring system;
- Selection of the textile system;
- Embedding of the sensors in the textile system through embroidery.

### 2.2. Sensors and Measuring System

Of the three possible options for choosing the type of textile, sensor-resistive, capacitive and piezo-resistive–capacitive sensors have been found to be the most suitable for the design of the e-textile. The capacitive sensors have low power consumption, high accuracy and a lack of requirements for special equipment and operating conditions. At the same time, the capacitive sensors are affected by environmental conditions: temperature and humidity. That is why the MPR121 controller of the capacitive sensors was chosen. The controller allows continuous and independent calibration of the inputs of each electrode, i.e., the current data obtained are compared with a base value that changes based on the variation of the background capacity. In addition, the data sampling rate was 64 ms, and its sensitivity was high, which significantly improves the capabilities of the filter system. It is possible to separate each electrode’s touch and release thresholds, which ensures independence from hysteresis.

**Figure 1:**
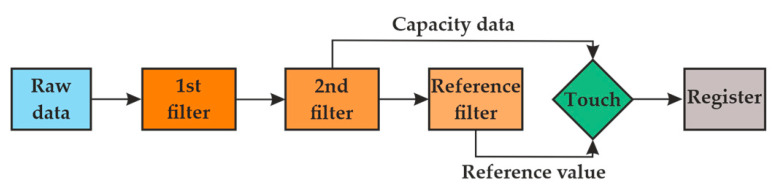
Data flow in the MPR121 capacitive sensor controller.

Figure 1 illustrates the data flow in the MPR121 capacitive sensors controller. The raw data outputs run through 3 levels of digital filtering to remove the encountered high- and low-frequency noises. After the first and second filtering, the result was the immediate capacitance of each sensing input. The reference value represents the capacitance variation over a long period caused by environmental changes such as atmospheric moisture and dirt. The data from the 2nd filter and the reference value were compared, and then the measured value was presented.

The number and location of the sensors were determined according to the critical areas of the human body where decubitus ulcers occur. They are found in the neck, shoulders, elbow, pelvis, thigh, legs and heel [17]. Therefore, when designing the sensor array, it was not necessary to fill the entire area of the mat regularly. The location of the sensors could be tailored to the anatomical features of the human body.

**Figure 2:**
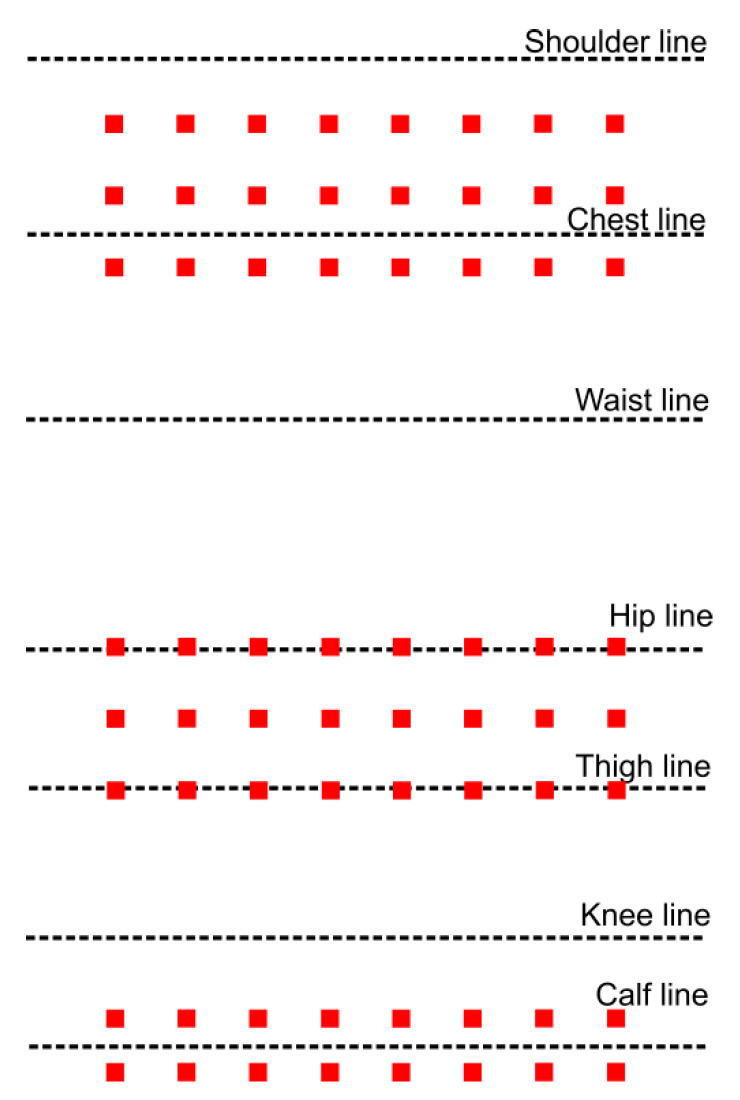
Scheme of the sensors’ arrangement.

Three zones were built in the designed e-textile mat following this strategy and the sizes of the most common human figures (Figure 2). The first two zones contained three rows of sensors, and the third-two rows. Thus, the designed prototype could be used by individuals with a different build. The zones with three rows of sensors were located along the lines of the back and hips. The zone with two rows was along the line of the calves. The width of the e-textile was 700 mm, which can be used in a single bed.

**Figure 3:**
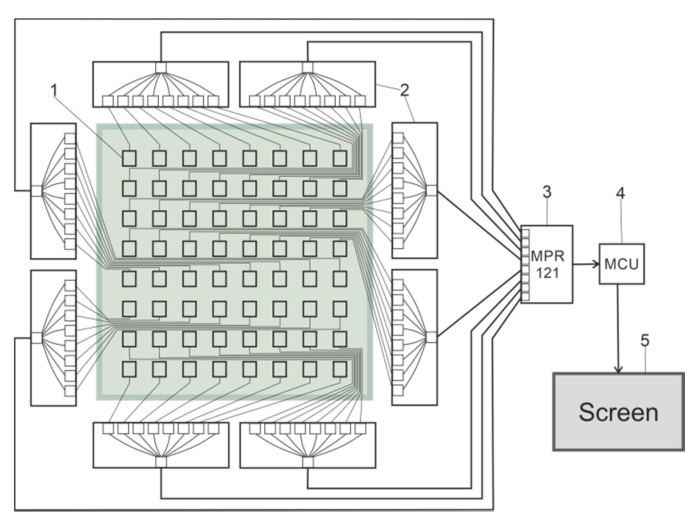
Scheme of the measuring system: 1—sensors, 2—multiplexers, 3—controller, 4—microcontroller, and 5—screen.

Figure 3 shows a diagram of the designed measuring system, which consisted of textile sensors (1), multiplexers (2), a controller (3), a microcontroller (4) and a screen (5). The need to include multiplexers was due to the large group of sensors which have data that must be collected and processed simultaneously in real time.

The multiplexer has several inputs and one output. It acts as a circuit breaker, where the connection is not mechanical, but is made through an integrated semiconductor circuit.

The entire development kit consists of a 12 bit ADC, Raspberry Pi 4 and 5” display. The first module was the Pi HAT version of the Adafruit MPR121 capacitive sensor. It measured 65 × 56 mm^2^ and had 12 sensor channels. It can be mounted on a Raspberry Pi 4.

The Raspberry Pi 4 minicomputer with dimensions of 85 × 56 mm^2^ works with an ARM processor. It has 2 GB of memory and multiple interfaces: 2 × micro-HDMI, TV/Audio OUT, 300 mbps ethernet, dual-band Wi-Fi, Bluetooth 5, micro-SD, 2 × USB 3.0, 2 × USB 2.0, over 20 GPIO ports, I2C, SPI, UART, I2S, CSI, DSI, and USB 3.0 ports and is powered by 5 V.

Connecting a display to the minicomputer makes it easier for the user to monitor the results of the measuring system. It is compatible with Raspberry Pi 4.

### 2.3. The Textile Phase

The textile system, which plays the role of a carrier phase, can be developed from layers of different types and thicknesses, which can vary in number. The most important properties for the upper layer of the system are high wear resistance, low elongation under tensile load, good air permeability, lack of peeling and the possibility of trouble-free machine embroidery. The woven macrostructures had better performance than the knitted macrostructures in terms of low elongation under tensile load. Therefore, a woven fabric of 100% cotton with mass per unit area of 230 g/m^2^ was chosen for the e-textile mat.

The requirements for the second layer were good air permeability, good absorption and low cost.

### 2.4. Embedding of the Sensors

#### 2.4.1. Machine Embroidering

**Figure 4:**
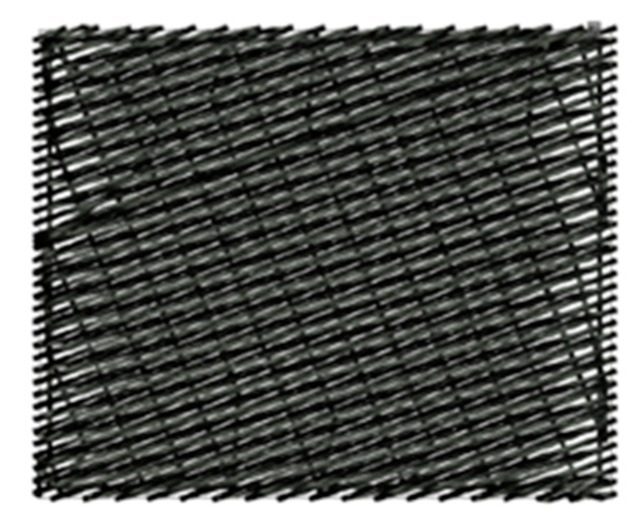
Object with a weave stitch line.

From the analysis of the motifs applied so far for developing resistive and capacitive textile sensors by machine embroidery [6,7,8,12,13], it was found that the motif’s shape was usually rectangular, filled with weave stitch line. The weave stitch line (Figure 4) is used in objects where covering stitches are needed or in combination with other types of underlay stitches. Only the authors of a recent publication [13] studied five motifs in which the distance between the electrodes was constantly preserved and the length of the conductive thread was significantly reduced.

The machine embroidery of the sensors was made with an MB4 Janome machine with one head and four needles. Madeira Germany conductive HC 12 thread was used for the upper and lower threads. HC 12 is a twisted polyester polyfilament with silver coating, a linear density of 235 × 2 dtex and an electrical resistance < 100 Ω/m. The main disadvantage of this thread is its durability, which according to [18], is approximately 10 washing cycles. The best performance had the stainless-steel microfibre thread, but there were difficulties with its application in machine embroidery.

A crucial feature of the embroidery of electrically conductive threads is that the work process is not interrupted due to the fact of thread break; otherwise, the electrical circuit also breaks. It is also essential to avoid the accumulation of stitches on top of each other.

The test samples with the embroidered sensors were made with a woven macrostructure in a twill weave (120 g/m^2^ mass per unit area) using a non-woven macrostructure on the backside (70 g/m^2^ mass per unit area).

#### 2.4.2. Electrical Resistance and Capacitance Measuring

**Figure 5:**
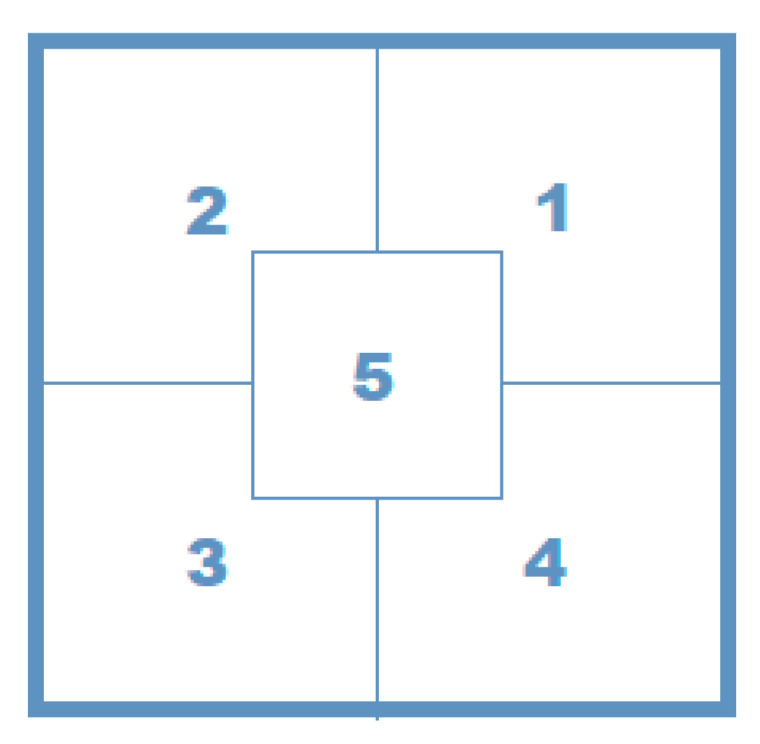
Scheme for the electrical resistance measurement of the embroidered element: starting in zone 1 and finishing in zone 5.

The measurement of the electrical resistance and capacitance was conducted with a digital LCR-819 m of GW Instek with an accuracy of 0.05%, capacity range from 0.00001 pF to 99.999 nF, electrical resistance ranging from 0.00001 to 99.999 Ω, and measurement speed of 68 ms. The resistance was determined in five zones (1 to 5) of the embroidered element according to the scheme in Figure 5.

**Figure 6:**
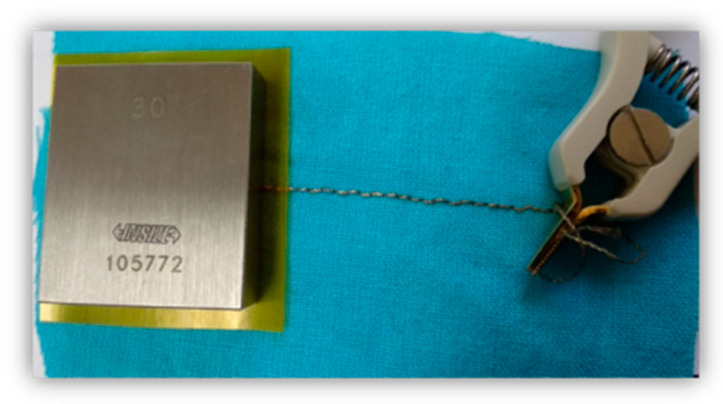
Scheme for the capacitance measurement.

The experimental scheme for the capacitance measurement is presented in Figure 6. The first electrode was the embroidered sensor, and the second was a flat parallel end measure made of stainless steel with dimensions 30 × 32 × 8 mm^3^. A standard test was performed at a voltage of 1 V and a frequency of 1 kHz.

## 3. Results and Discussion

### 3.1. Selection of the Textile Sensors

When incorporating the sensors using embroidery with conductive threads, it is crucial to follow specific rules:

- Avoid overlapping stitches;
- Minimum thread length;
- Making the motif without breaking/cutting the threads.

**Figure 7:**
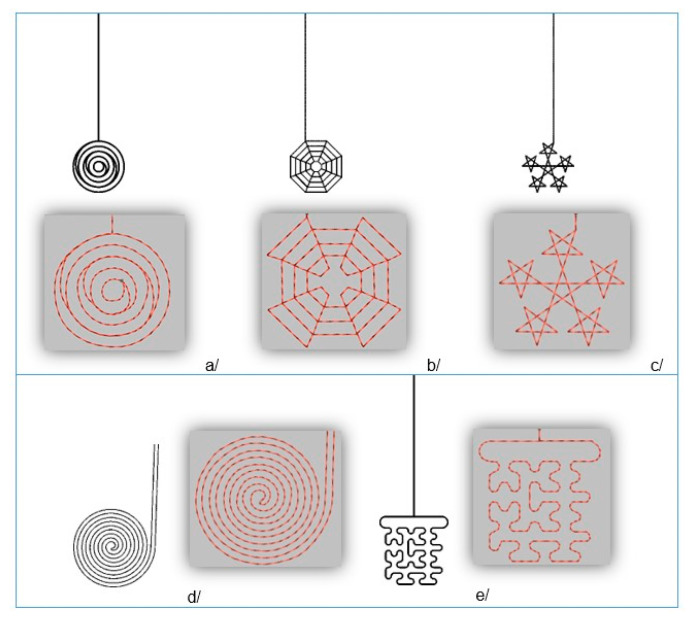
Design of the sensor patterns: (**a**) concentric circles; (**b**) cobweb; (**c**) five-pointed star; (**d**) spiral; (**e**) Hilbert curve.

Five motifs were developed to incorporate the sensors in the textile systems: concentric circles, cobweb, spiral, five-pointed star and Hilbert curve. They were designed based on known mathematical functions and fractals and observed work conditions with conductive threads. These variants have not been proposed in the literature yet. The sensor designs were made with 3D modelling software, after which the embroideries were created with Digitizer MB V 3.0 (Figure 7).

**Figure 8:**
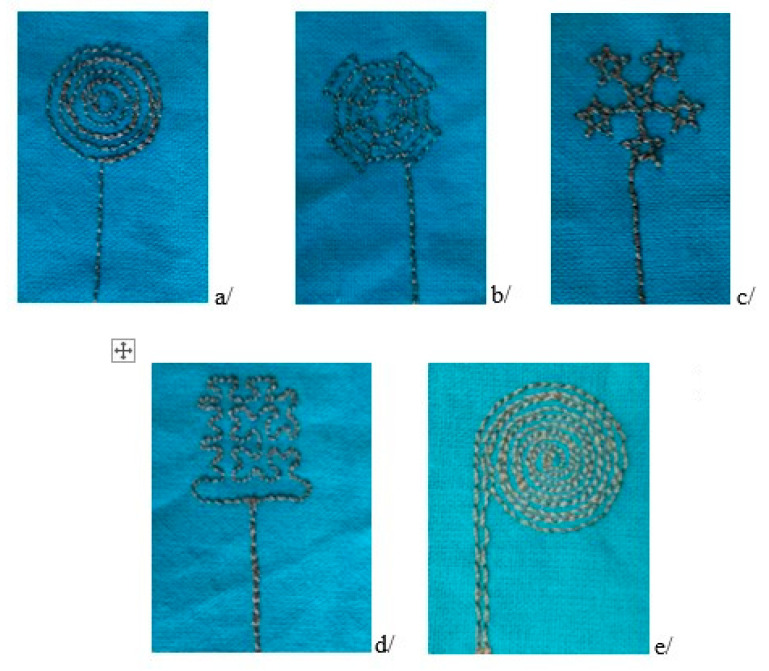
Embroidered textile sensors: (**a**) concentric circles; (**b**) cobweb; (**c**) five-pointed star; (**d**) Hilbert curve; (**e**) spiral.

All motifs were designed with the same overall size of 20 mm, which is in full accordance with the literature. Straight double-sided stitch line, which consumes less thread length, was used, with a stitch step of 2 mm. The produced samples with the sensors are presented in Figure 8. It resulted that only the motif with concentric circles cannot be made without overlapping the stitches, which is undesirable.

The results of the measurement of the sensors’ electrical resistance and capacitance in different patterns are shown in Table 1. The cobweb and five-pointed star were the motifs with the lowest electrical resistance. The spiral and cobweb motifs showed the highest capacitance, approximately 20 pF. Since the cobweb motif had a lower electrical resistance and a lower conductive thread consumption, it was selected for the e-textile mat design.

### 3.2. Production of the Textile Sensors

**Figure 9:**
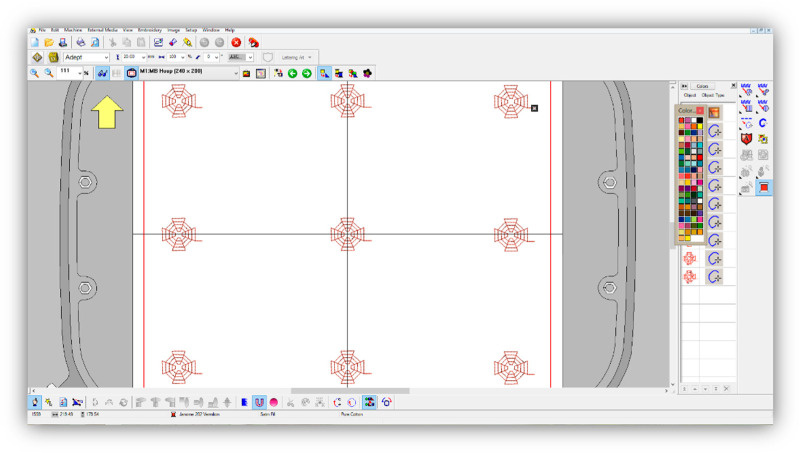
The development of a program for the sensors’ incorporation with Digitizer MB.

The sensors’ incorporation through embroidery required marking the lines on the fabric along which the sensors would be located. Thus, it was possible to centre the textile system in the embroidery frame. With the application of an embroidery frame with the largest dimensions, it was possible to digitise and produce a total of nine sensors simultaneously. The group of nine sensors had to be centred relative to the zero in the *x*- and *y*-directions (Figure 9).

**Figure 10:**
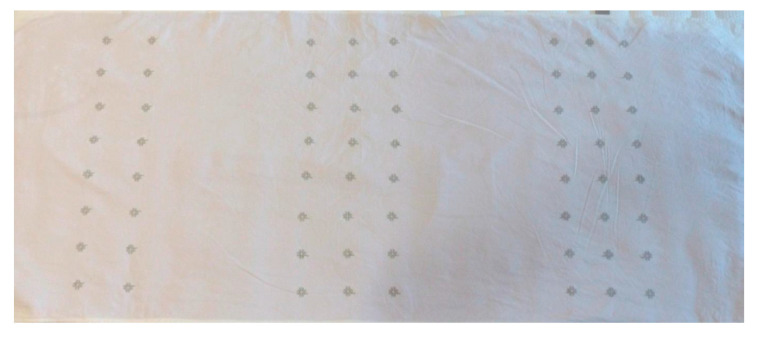
The e-textile mat with the embroidered textile sensors.

Figure 10 presents the embroidered textile sensors for body position. Because the photo was taken at an angle, the rows of embroidered image sensors seem to be located at an angle, resulting in image distortion.

**Figure 11:**
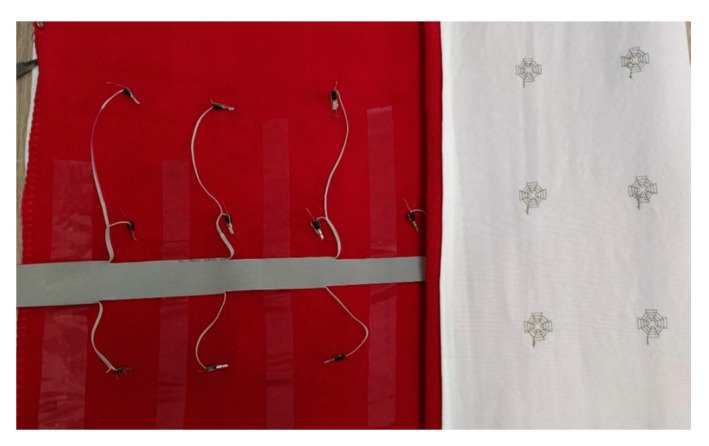
The reverse side of the e-textile mat with the embroidered textile sensors.

When incorporating the sensors, a certain length of the upper thread (approximately 15–20 cm) was left free to be connected with the flat ribbon cable passing from the reverse side of the mat. The ribbon cable passed through the entire width of the product, ensuring that an equal length of conductive thread was used for each sensor. Figure 11 shows an image of the connected sensors with the flat ribbon cable using a cable lug. The width of the ribbon cable was consistent with the number of sensors in the three groups. The application of a second layer in the mat ensured the preservation of the comfort and stability of the product (to avoid the mat’s wrinkle when the body turns) during operation. An ethylene-vinyl acetate (EVA) foam could also be used for a second mat layer.

### 3.3. The Software Connection

Part of the project was developing specific software (application) for control of the multiplexers, collecting the data from the controller of the capacitive sensors and sending them in a protocol on a serial port. The application was created in the processing environment for visualisation of the results. A more straightforward interface was offered to simplify the data reading by the user. A mesh of squares (visualising the sensors) was used, which changed from black for the passive sensors to light green for the active. The depth of the green colour varied according to the change in the capacitance, which represented the pressure that the person exerted on the sensors. Therefore, it shows that the air gap between the body and the sensors decreased or the overlapping area increased. This way, the user (caregiver, medical staff) could be informed about the bedridden person’s position, which had not been changed.

**Figure 12:**
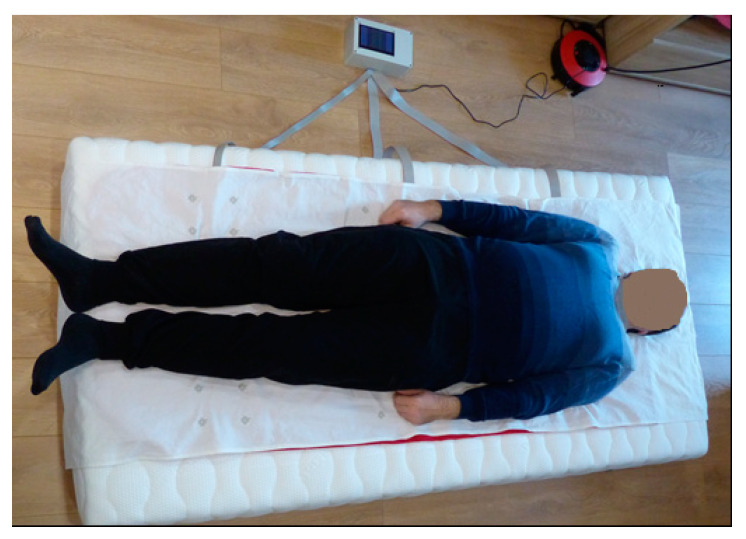
The experimental setup for the measurement with the e-textile mat.

To verify the data obtained from the sensors, experimental studies were performed with an individual in two postures, essential for the human body in a supine position: posture one, on the back (Figure 12), and posture two, in a sideways pose.

**Figure 13:**
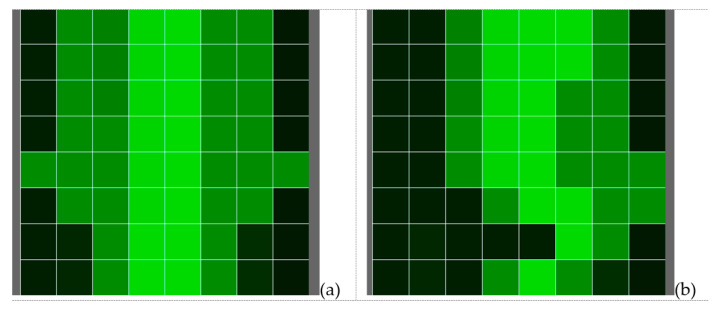
Results from the measurements: (**a**) back posture; (**b**) sideways posture.

The sensor readings were displayed on the screen of the Raspberry Pi 4 minicomputer. The squares with the darkest colour corresponded to the passive sensors, and those with lighter ones to the activated ones. The motives that were obtained in the two postures of the human body are presented in Figure 13.

The software makes it possible to set a time interval for which the sensors move from one shade to another. Thus, the user could determine whether the bedridden person has performed movements of the torso and lower and upper limbs. At this stage, it is impossible to accurately determine the pressure values in the separate zones, as the capacitive sensors are influenced not only by the hardness of the mattress used for the test but also by the environmental conditions. However, the presence of reference values of the sensor controller before each measurement eliminates the influence of the environment.

## 4. Conclusions

The design and development of a prototype of an e-textile mat with textile sensors for avoiding decubitus ulcers in bedridden persons were presented. The developed monitoring system and software allowed real-time monitoring of the body position. The sensor was produced with silver-coated conductive thread, which has a good sewability.

Five variants of the pattern for the textile sensor were developed, and their capacitance was measured. The best performance had the design with cobweb, with a capacitance of 19.28 pF, and it was used for the smart mat production.

Future work in this direction could be expanded by including a group of sensors (at least two rows of three sensors each) in the head area. It is also possible to look for other software solutions for visualisation of the results

We hope that our work will encourage the development of similar devices in the field, which would increase the comfort of the bedridden persons and help their caregivers’ performance.

## Figures and Tables

**Table 1:** Electrical resistance and capacitance of the sensors in different embroidery patterns.

Pattern		Concentric Circles	Cobweb	Five-Pointed Star	Spiral	Hilbert Curve
Electrical resistance, Ω	Average	3.95	1.65	1.43	2.49	11.28
	Standard deviation	0.34	0.20	0.13	0.95	1.25
Capacitance, pF	Average	15.58	19.28	15.76	25.68	17.5
	Standard deviation	0.492	0.207	0.285	0.312	0.303
Invested thread length, m		1.06	1.10	0.98	1.49	1.06
Capacitance/thread length		14.70	17.53	16.08	17.23	16.51

## Data Availability

Not applicable.
